# Knowledge gaps on paediatric respiratory infections in Morocco, Northern Africa

**DOI:** 10.1186/s13690-015-0076-x

**Published:** 2015-06-15

**Authors:** Imane Jroundi, Chafiq Mahraoui, Rachid Benmessaoud, Cinta Moraleda, BadrSououd Benjelloun, Quique Bassat

**Affiliations:** ISGlobal, Barcelona. Ctr International Health Research (CRESIB), Hospital clinic. Universitat de Barcelona, Calle Rosselló 132, 4°, PC 08036 Barcelona, Spain; Laboratoire de santé publique et de médecine communautaire. Faculté de Médecine et de Pharmacie de Rabat, Université Mohamed V, Rabat, Morocco. AV Mohamed Belarbi El Alaoui Rabat Institut, BP 6203 Rabat, Morocco; Hôpital d’Enfants de Rabat (HER), Centre Hospitalier Universitaire Ibn Sina, Rabat, Morocco. Rue Lamfadel Cherkaoui Rabat Institut, BP 6527 Rabat, Morocco

**Keywords:** Acute respiratory infection, Pneumonia, Morocco, Children under five, Burden, Epidemiology, Respiratory syncytial virus, Bacteria

## Abstract

**Background:**

The burden of acute respiratory infections (ARI) among Moroccan children remains significant. However, scarce information is available regarding trends in its epidemiology and etiology, or regarding its associated prognostic factors.

The purpose of this work was to review available data on the burden of ARI among children under five years of age in Morocco.

**Methods:**

A systematic review was conducted for the period 1997–2014 using the PRISMA proposed methodology. Various online databases were screened, in addition to physical libraries of Moroccan medical schools, and official reports of the Moroccan Ministry of Health. Search queries in English and French languages included: Respiratory Tract Infections, pneumonia, epidemiology, etiology, microbiology, mortality and Morocco. The documents were included for analysis when they reported original data on the incidence, distribution, or a clinical description of the diseases or their etiology or described clinical management or national preventive strategies.

**Results:**

Thirty-two documents were included in the final analysis. 21 of which had been published. In 2012, ARI caused 13% of paediatric deaths, half of the consultations at health facilities and third of the paediatric admissions. The microorganisms more frequently identified among hospitalized children were *Streptococcus pneumoniae* (38%) and *Haemophilus influenza type b* (Hib) (15%). The MOH introduced Hib vaccines into the national immunization program (PNI) in 2007and the 13-valent vaccine against pneumococcus in 2010. The national first line antibiotics recommended for non-severe ambulatory treatment is Amoxicillin. Studies of antibiotic resistance showed from 1998 to 2008 a 22% increase in the rate of penicillin non-susceptibility among *Streptococcus pneumoniae* isolates. Viral respiratory infections and the role attributed to air pollution in the incidence of ARI have been poorly characterized.

**Conclusions:**

Further efforts should be made towards the development of adequate surveillance programs to better clarify the epidemiology, etiology, antimicrobial susceptibility patterns and the effectiveness of the preventives and curatives strategies in place against paediatric ARIs in Morocco. Additionally, a holistical approach should be used to identify the heath determinants of ARIs among children.

## Background

Current estimates confirm that around 15% of global deaths in children under the age of 5 are caused by pneumonia [1,2]. This is equivalent to almost one million annual child deaths, making it the single biggest killer of children worldwide. However, some 451 000 lives have been saved in the last decade, thanks to the protection of children from indoor pollution, the promotion of breast feeding and hand washing and to the intensified worldwide efforts to increase access to effective vaccination and treatment management. Despite this, the high toll imposed by pneumonia remains intolerable. Reducing the burden of pneumonia mortality is essential to achieve the fourth Millennium Development Goal (MDG) of reducing child mortality. To assess how national programs are progressing towards the reduction of the disease burden, it appears imperative to establish adequate surveillance systems capable of: 1) Measuring this burden; 2) Describing its epidemiological trends and 3) assessing in the detail the underlying etiologies and pathogens involved [3].

However, most of the developing countries with high burden of morbidity and mortality associated to ARI still lack the necessary data on the real burden, epidemiology and etiology of ARI within their territories [4,5]. Such knowledge gaps seriously hinder the capacity of those countries to evaluate the progress on their preventive strategies in the fight against pneumonia, or the trend on antimicrobial sensitivity, which should guide their treatment policies [6,7]. Indeed, in most of these countries the estimation of the burden of ARI is based on specific surveys at the national or regional level, or more isolated results deriving from hospital records [6,7].

In Morocco, a middle-income country in the North of Africa, pneumonia in children under five years of age, remains a major public health challenge [2,8]. The Moroccan Ministry of Health, promotes the integration of multiple strategies to fight against pneumonia [9,10]. Its pneumonia specific policies encourage exclusive breastfeeding and hand washing. At the peripheral level, Morocco uses the integrated management of childhood illnesses approach for the early recognition of pneumonia and is adequate management [11]. Beside this, the Moroccan Ministry of Health (MOH) introduced in 2007 and in 2011 the Hib and the pneumococcal (13-valent) vaccines, and further trained its health professionals in the adequate management of pneumonia cases.

In spite of these strategies, both the MOH and the United Nations (UN) still report that the burden of ARI among Moroccan children remains ostensibly important despite progresses in the implementation of better treatment and preventive strategies [2]. Presumably, the lack of adequate updated data on the real burden, etiology and relevance of pneumonia is an important barrier against the implementation of better evidence-based measures designed to lower the toll associated to respiratory infections.

In this respect, determinants of respiratory disease in Morocco, are can be seen in many other places, remain multifactorial [12]. Besides the individual determinants of infection (pathogen based and human host characteristics, such as age, gender and other risk factors such as prematurity, nutritional status and breastfeeding), other determinants such as environmental exposure (atmospheric pollution and other kinds of indoor pollution, such as smoke, social determinants (including parental educational and the socio economical level), may all play an important role in the epidemiology and the burden of ARIs in Morocco.

In order to evaluate the current knowledge gaps regarding paediatric ARIs In Morocco, we reviewed several databases (online and in medical school libraries) in search for relevant publications for the past 17 years, aiming to identify knowledge, gaps, and challenges ahead in terms of the prevention, diagnosis and treatment policies for ARI. We deliberately chose this 17 year long period because it coincides with the start of the ARI control program in Morocco and the implementation of the IMCI program in Morocco.

## Methods

**Figure 1:**
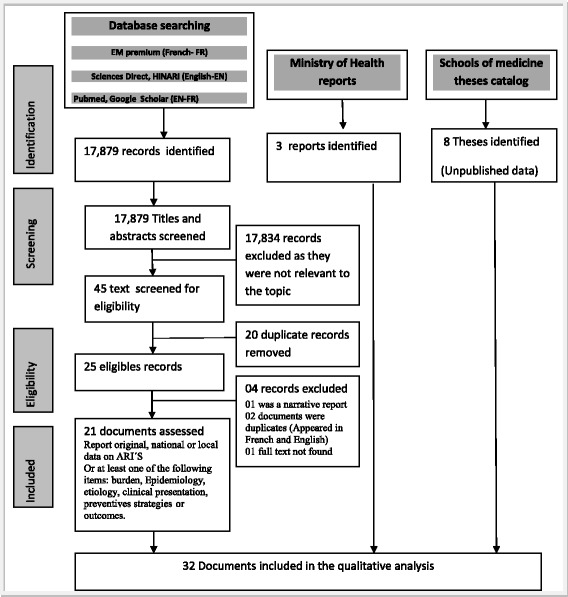
Flow diagram for the extraction of the records related to ARI in Morocco among children published from 1996 to 2014.

A systematic review was conducted using the PRISMA proposed methodology [13]. Pubmed®, Google Scholar®, Hinari®, Sciences Direct®, EM Premium® online databases were screened, in addition to physical libraries of three Moroccan medical schools (Rabat, Casablanca and Fez) in search of unpublished PHD theses. Finally, official reports of the Moroccan Ministry of Health (MOH) were also searched and publications from 1997 to 2014 reviewed in both French and English languages. The search query terms used included: (“Severe Acute Respiratory Infections/epidemiology” OR “Severe Acute Respiratory Infections/etiology” OR “Severe Acute Respiratory Infections/microbiology” OR “Severe Acute Respiratory Infections/mortality” OR “pneumonia”) AND “Morocco”). The data found were identified and selected by two readers: a public health professional and a paediatrician using the PRISMA flow chart. (Figure 1 summarizes the flow diagram). After the screening, the two readers merged the documents to study their eligibility using items from the PRISMA checklist (title, objective, study population, study setting, information sources, process of data collection, and outcome measures) [13]. Then, they made consensus decision about the inclusion of the eligible documents in the final analysis. The studies inclusion’s criteria were: reporting original data on the incidence, distribution, or a clinical description of the diseases or their etiology or describing medical treatment or national preventive strategies. Studies were excluded if the documents implied duplication or when, full texts of abstracts were not found.

### Results and discussion

This first screening yielded seventeen original articles published in peer review journals, 12 in English language and five in French, one original paper was published in French language in a Moroccan peer reviewed journal. Four abstracts were published in French language and two in English. Finally three additional MOH reports were found related to the topic. From the school of medicine libraries’, eight unpublished doctoral theses were extracted. After analysis of each of those publications, two reporting duplicate results were excluded and one was found to be not eligible, as it did not report any original result.

These documents describe paediatric ARI-related surveys in Morocco conducted in the last 17 years. The data could be classified according to the following three groups: 1) those documents describing severe pneumonia cases admitted to the university hospitals (7 published, 8 unpublished). 2) Those describing samples obtained from pneumonia patients and analyzed by laboratories of university hospitals (10, all published), and finally, 3) National data provided from the annual statistics report from the MOH including a survey evaluating the IMCI program (one study, published) and a study conducted within the community (three studies, all published).

#### Description of national health statistics

**Figure 2:**
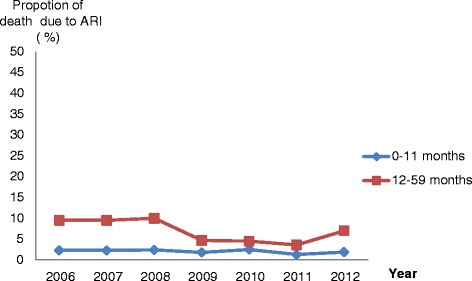
Proportion of under five mortality due to ARI by age group from 2005 to 2012. (Data extracted from the National Health Statistics annual reports and estimated from death certificates).

The World Health Organization (WHO) reports that ARI remains in Morocco the leading individual cause of under five mortality, accounting for a total of 13% of all deaths in this age group in 2012 [2]. Figure 2 illustrates the proportion of annual deaths among under five according to age group (infant and older children) which can be attributable to ARIs, from the period 2005 to 2012. Data show an important decrease of ARI-associated deaths from 2008 to 2011 among older children. No changes can be observed for infants during the same period. No differences in the distribution of death gender were evidenced (data not shown). WHO has also estimated that in the year 2012, only half of the children with suspected pneumonia were taken to an appropriate health provider, and only 49% suffering from pneumonia got antibiotics [2].

**Figure 3:**
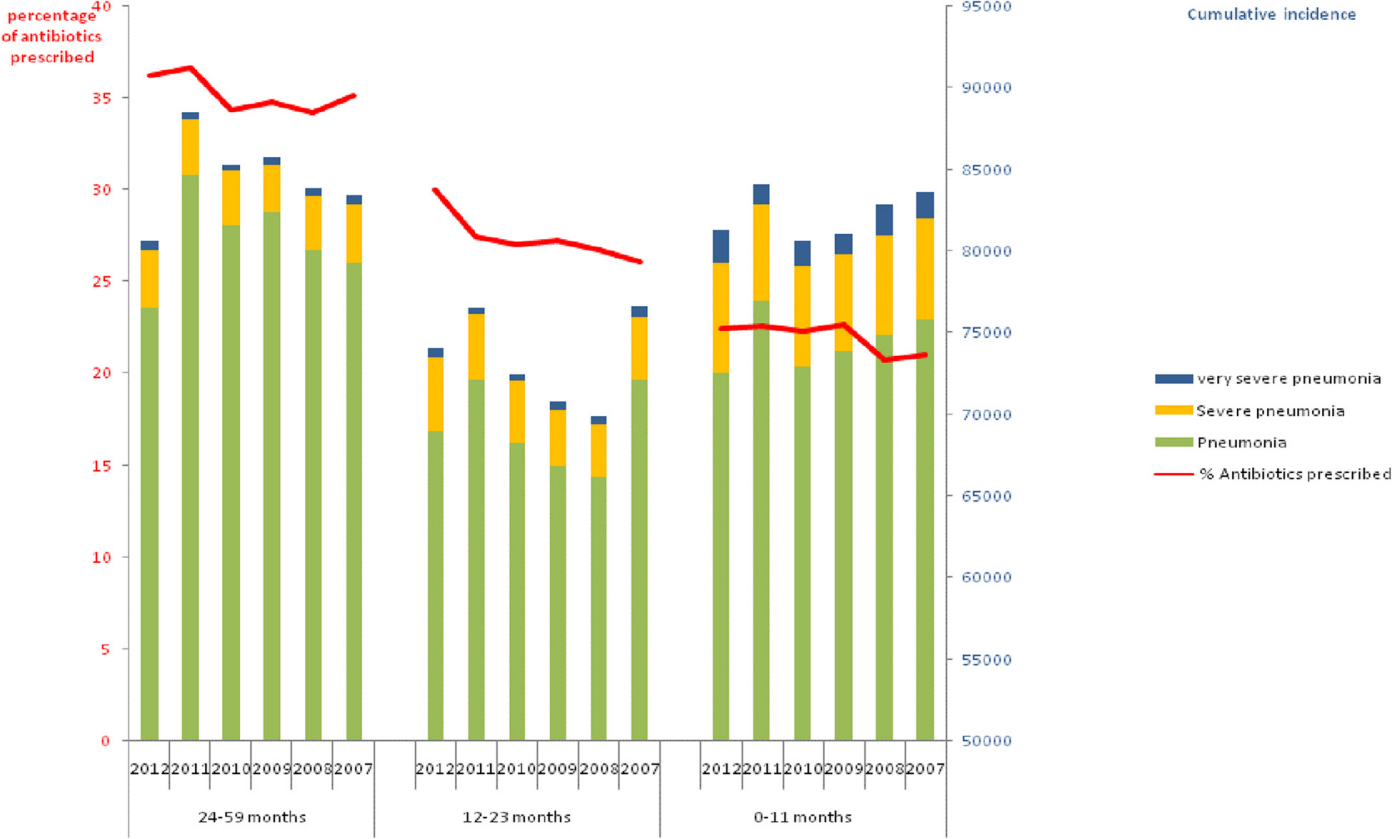
Evolution of the number of outpatient visits (per age group) to primary health care centers for acute respiratory infections (ARI) and the proportion of those visits being prescribed antibiotics among children under five years old at the national level from 2007 to 2012. Data extracted from the National Health Statistics annual reports.

The summarized data from 2007–2012 obtained from Morocco’s National annual health statistics (Figure 3), show that the most commonly notified types of ARI both in urban and rural areas are pneumonia cases, followed by severe pneumonia cases. The very severe cases are less prevalent. The most commonly affected age group included toddlers age 24–59 months. But the most severe cases occurred in a higher proportion among infants (0–11 months). The figure also shows the proportion of ARI cases which subsequently were prescribed antibiotics, with an increasing trend in all age groups throughout the study period.

**Figure 4:**
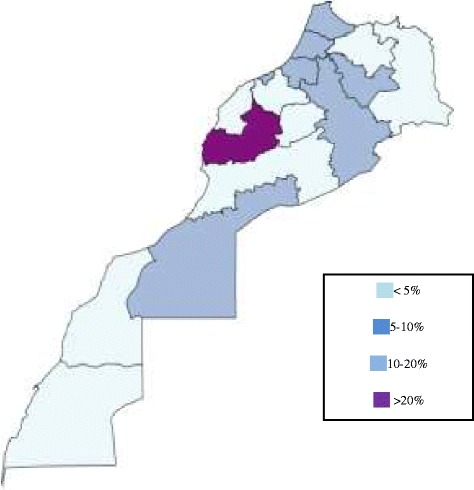
Map of Moroccan regions for which incidence of pediatric ARI have been summarized for the year 2012. Data extracted from the National Health Statistics annual report.

Figure 4, shows the distribution of the incidence of ARI for the different country regions for which data are available. Almost 54% of all ARIs are reported from rural areas, whereas the remaining (46.4%) occur in urban setting.

In the places where the IMCI program has been implemented, there are some data collected from the MOH since 1997. The indicators collected estimate the performance of trained nurses in the diagnosis of pneumonia (whether these episodes diagnosed were severe or not), and the proportion of those receiving antibiotics. A study of the quality of IMCI six months after its implementation in two Moroccan regions as a pilot project showed that following the IMCI program allowed a better quality of care, measured by better indicators of correct antibiotic prescription, and a better overall classification of the severity of illness [11].

#### Clinical trends and characteristics of ARI in Morocco from 1997 to 2014

Different studies were found describing the clinical characteristics of patients (range 7–700) admitted to public paediatric hospitals, including clinical patterns of ARI, duration of hospitalization, treatments received and outcomes. The reported observational period in each of these studies ranged from one to ten years. What emerges from these studies is that: the majorities were descriptive studies; only one being a comparative study and using statistical analysis. Study populations were also quite distinct, including in certain studies hospitalized pneumonia cases only, whereas in others, outpatients with ARI. The inclusion criteria were based on the clinical case definition, or on radiological or microbiological criteria. Age at recruitment in these different paediatric studies ranged from a minimum of two months to up to 15 years of age. Most of these studies, have never been published and are available as doctoral theses, and only one reports the bacteriological evidence of the causative organism of ARI in half of its studied cases (5/10= 50%). Table 1, summireses the information collected through these unpublished studies.

**Table 1:** **Unpublished sources of information on ARI in children in Morocco: from 1997 to 2014**

**Year**	**Place**	**Source**	**Study population**	**Available information**
1999	Rabat Paediatric hospital	Thesis, faculty of medicine of Rabat	Admitted patients with bronchopneumonia	Retrospective data: clinical, radiological, microbiological and outcome of patients.
1999	Rabat Paediatric hospital	Thesis, faculty of medicine of Rabat	Inpatients and out patients with broncholalveolitis	Retrospective data: clinical, radiological and outcome of patients
2000	Rabat Paediatric hospital	Thesis, faculty of medicine of Rabat	Inpatients and out patients with broncholalveolitis	Retrospective data: clinical, radiological and outcome of patients.
2003	University hospital of Casablanca	Thesis, faculty of medicine of Casablanca	Isolates among children admitted for invasive pneumococcal disease	Case study
2007	Rabat Paediatric hospital	Thesis, faculty of medicine of Rabat	Inpatients with first episode of broncholalveolitis	Retrospective data: clinical, radiological and outcomes of patients
2008	University hospital of Casablanca	Thesis, faculty of medicine of Casablanca	Inpatients with pleural effusion	Retrospective data: clinical, radiological, microbiological and outcome of patients.
2009	University hospital of Casablanca	Thesis, faculty of medicine of Casablanca	patients admitted for acute community pneumoniae	Retrospective data: clinical, radiological, microbiological And outcome of patients.
2011	University hospital of Casablanca	Thesis, faculty of medicine of Casablanca	Patients admitted for bronchoalveolitis	Etiologies of bronchoalveolitis.No virological investigation.

The clinical patterns of ARI throughout the past 17 years have varied in the different studies available. For instance, studies looking at acute lobar pneumonia and pleural effusion are common from 1998 onwards. However, a thesis produced at the university hospital of Fez, reported 53 cases of purulent pleurisy between 2006 and 2009 [14]. Studies conducted between 1994 and 1998 show that the frequency of viral bronchoalveolitis accounted for 10% of cases of ARI admitted to university hospital. Patients with acute lobar pneumonia and bronchopneumonia represented at that time 38% and 40% of the ARI respectively. However, a recent study [15] reported that among 700 patients admitted to a paediatric ward in Rabat, fulfilling WHO clinical severe pneumonia case definition, only 28% of them had a clinical diagnosis of suspected bacterial pneumonia (as given by the clinician in charge) at the time of discharge. The same study reported that the nasopharyngeal carriage of respiratory virus was almost 92% and that invasive bacterial disease was identified only in 2% of patient [15], in spite of universal blood culture and PCR investigation of invasive bacterial diseases screening among all study participants (n=700). Beside this, and from year 2000 onwards, the information available from University Hospital of Rabat, has only focused on cases of viral bronchoalveolitis. Conversely, the reports coming from Hospital of Casablanca, presented nine blood culture confirmed cases of pneumococcal community-acquired pneumonia and 145 further pneumonia cases without bacteriological confirmation during an observation period of two years. A further case of pneumonia associated to pleural effusion secondary to *Streptococcus Pneumoniae* was reported in a one year-long survey. In 2000, ARIs accounted for 30% of all emergency consultations and 20% of the total number of admissions in the paediatric infectious diseases ward. [16] The most affected groups of age were infants and children less than 2 years of age, representing up to 75% of all paediatrics patients admitted for ARI. This distribution did not seem to change across the 17 years, since a published study in 2014 conducted in the same ward showed similar findings, and reported that almost 30% of children attending the paediatric emergency department had respiratory symptoms, and that 42% of those would be eventually admitted [15].

In terms of seasonality, ARI’s admissions for bronchiolitis episodes usually peaked during the winter season, whereas pneumonia and bronchitis are distributed throughout the year [15]. The most consistent clinical feature of pneumonia described in these studies during the 17 years long period of assessment included respiratory distress, fever and cough. The average length of stay ranged usually from two to five days and the associated in-hospital case fatality rates from one to five percent. Only one study [15], documented the nutritional status of children admitted for ARI, and showed that 5% of these cases occurred in severe malnourished children (according to a weight for-age Z score WAZ < 3- SD).

#### Description of laboratory data

Morocco lacks a national established system for the monitoring of antimicrobial resistance of pathogens causing ARI. However, there is some information provided by laboratory university hospitals. During the period 1994 to 1998, most isolated germs included in order of frequency *Staphylococcus aureus*, followed by *Streptococcus pneumoniae* and H*aemophilus influenzae* type b. These pathogens were isolated from blood or pleural fluid. The potential role played by viruses in the etiology of pneumonia was not at all explored before the epidemic episodes of influenza. Few published studies reported the clinical features of the epidemic episodes of influenza H1N1 in 2009 [17,18], and showed that children had the highest proportion of laboratory-confirmed influenza through the influenza sentinel surveillance system. Data on other virus (*Respiratory syncitial virus (RSV), parainfluenza,* 1,2,3 and *Adenovirus*) explored through this sentinel surveillance are still not available [19].

More recently in 2014, data from a paediatric university hospital reported that the invasive pneumococcal disease was infrequent, but the nasal and nasopharyngeal carriage of *S. pneumonia* was common. [15] In this same study, a full panel of respiratory viruses was screened among all study participants (n=700), yielding extremely high (>90%) nasopharyngeal mono or multiples infection rates. Most commonly identified viruses included Rhinovirus followed by respiratory syncytial virus (RSV) and Adenovirus. Importantly, Human metapneumovirus was also frequently detected and the only of those viral pathogens independently and significantly associated with an adverse outcome [15].

Regarding the monitoring of antimicrobial susceptibility, some data are available for *Streptoccoccus pneumoniae*. Active laboratory surveillance was held from 1994 onwards in the microbiology laboratory of University Hospital of Casablanca, including all the strains collected from the wards (adult and paediatric) of three university hospitals located in Casablanca [20]. Among 1152 strains collected over these 14 years of ongoing surveillance, antibiotic susceptibility for this pathogen decreased significantly, and importantly, antibiotic resistance was more prevalent among isolates from children. From 1994–1997, 12.5% of the strains were found to have reduced susceptibility to penicillin, this figure increasing to 15. 3% from 1998 to 2001 [21], to 18. 9% from 2002 to 2005, and to 23. 5% from 2006–2008 [22,23]. This study included all types of strains. The same center had contributed in the collection of data within a regional network (ARMed) [24] during the period 2003 to 2005. Notably, Morocco’s *Streptococcus pneumoniae* isolates had one of the highest erythromycin resistances within the network. In the city of Rabat two independent surveys were conducted in two distinct university hospitals during the periods 1997 to 2001 and 2006 to 2007, also trying to assess the antibiotic susceptibility patterns of *Streptocococcus pneumoniae* [25] and other respiratory pathogens [26]. The first study showed a low susceptibility to beta-lactams (7,8%), but one of the resistant isolates had been isolated from the cerebrospinal fluid of an infant with meningitis secondary to meningeal breach [25]. The second study showed that 2/6 (33.3%) of the *pneumococci* were fully susceptible to amoxicillin, and 80% to erythromycin. 91 strains of *H. influenzae* type b were also obtained and investigated [27] with the vast majority (97, 9%) being susceptible to amoxicillin+/clavulanic acid, and the totality (100%) to cephalosporins. This study showed the lowest resistance rates to penicillin from *Streptococcus pneumoniae* observed in Northern Africa [24].

Samples collected in 2010 within a hospital setting, from patient’s under 5 years of age admitted for severe clinical pneumonia [28], showed that *S.pneumoniae* isolates had moderately low resistance rates to commonly available antibiotics (Erythromycin 20%, cotrimoxazole 24%, penicillin G 10% and amoxicillin 14.6% ) [28].

The surveillance of pneumococcal serotype distribution among admitted patients in Casablanca, before the introduction of the pneumococcal vaccine, showed that the most prevalent circulating serotypes were 19F, 14,6,18 and 9 [22]. The same distribution was also confirmed among healthy children, in whom the prevalence of nasopharyngeal carriage was 45, 8% [29]. The analysis of the carriers, suggested that carriage was more likely in children who had exclusively breastfed for less than two months, had a low socio.economic background, or lived in crowded houses [29]. The commonly identified serotypes included 19 F,6,14,23,18 and 9. In a study conducted in Rabat city [15], coinciding with the year of pneumococcal vaccine implementation, the most prevalent serotypes isolated among these admitted patients were 6A, 19F and 6B. The current 13-valent vaccine, introduced into the national immunization program in 2011, would cover up to 85% of isolates.

#### Clinical management of ARI

Outside the public sector, management of ARI in Morocco is difficult to document, due to the widespread availability of private practitioners’ in urban areas and some of rural areas in the country. In these ambulatory private settings there are no official documents which reflect the private physician’s practices. IMCI guidelines, which propose a standardized and documented approach to diagnosis and management, have not been properly assessed at national scale in Morocco, and thus, little information exists on how they are truly applied in the rural areas. Conversely, in university hospital settings, specific protocols for the management of ARI based on consensus exist and are followed.

A recent study conducted in the university hospital of Rabat [28], reports that before their admission, 30% of patients with ARI received antibiotics within the two weeks preceding their hospitalization. These antibiotics were prescribed in 86.5% of the cases by a physician. The most common antibiotics used prior to arrival to hospital, were Amoxicillin/Clavulanic acid, followed by amoxicillin and Macrolides. The same study documented that once admitted, these same patients often received in decreasing order of frequency, cephalosporines, macrolides and gentamicin. Most of these antibiotics were given parenterally.

#### Determinants, risk factors and prognostic of ARI

Specific data on independent risk factors for ARI are poorly described, and often never explored in the unpublished studies. However, infants and males appear to be consistently more at risk of being admitted as result of their ARI.

A recent survey [30], reported the risk factors of poor outcomes among children admitted for clinically severe pneumonia. The main independent risk factors for death or the requirement of the admission to the intensive care unit included prematurity, exposure to passive smoking at home, history of fever, the presence of cyanosis, pallor, ronchi at chest auscultation, unconsciousness on admission and human metapneumovirus infection. Other common risk/protective factors described in the literature (breastfeeding, age, malnutrition, parental and socio economical and educational level) were also explored in this study, but were not found to be associated in multivariate analysis with an adverse outcome.

One study was conducted within the community to assess the exposure of children to passive smoking and showed that around 34% of children participating were exposed at home [31]. Another study conducted in a hospital setting described slightly higher exposure rates (up to 40%) among children admitted with ARI [15].

The role of indoor pollution in relation to ARI was never explored. However, a single study exploring the air pollution as a determinant of asthma among schoolchildren in an industrialized city, was found and suggested that the children living in the most polluted industrial zone were most affected by respiratory diseases and, in particular, asthma and that the majority of the affected children were from underprivileged families and were malnourished [32].

Little anthropological research has been conducted in Morocco to assess whether family behaviors could affect or influence the management of ARI among children. A single study was done during 1998 in the region of Marrakech [33]. The study showed that the parental socio economical and educational level, and that access to the health system, or to transportation had an important influence in the parent’s behaviors. Illiterate parents predominantly preferred to start treating their sick children at home and herbal remedies were typically used. Additionally, visits to the traditional healers, which may also use herbs or more aggressive practices, including burning of the chest using a piece of burning charcoal through a blanket were also common. Such parents would only visit the physician as a last resource, when the previous methods had shown to fail, possibly coinciding with a worsening condition of their child. In Morocco, the abundance of a private medical sector has been related to an abuse or misuse of antibiotics, and to challenges related with the radiography examination of the chest among patients with suspected pneumonia, possibly in relation to high costs [33].

Available data reviewed regarding acute respiratory infections in Morocco in the past 17 years, offer a blurred picture (Table 2). Indeed, the scarcity of official published or unpublished data regarding the number one killer of children in Morocco is disquieting. Among the few available reports, consisting mostly of *ad hoc* descriptive studies from admitted patients to university hospitals, data are difficult to compare and to generalize, because the National Health Statistics do only represent the data collected from the public sectors. The private sector does not participate to the national health statistics, which cuts off the national statistics of important data, relating to the characteristics of the population attending private practices and to their care practices. Moreover, access to the health care system is mostly depending on the geographical characteristics of the regions and the contribution of the private health care sector in these areas. So, the distribution of the ARI in Morocco shown in figure 4, and the distribution of ARI between urban and rural areas, may not reflect the real burden and distribution of ARI within the country.

**Table 2:** **Keys knowledge gaps regarding paediatric ARIs in Morocco**

**Keys knowledge gaps regarding pediatric ARIs in Morocco**	
□	lack of consistent and homogeneous case definitions for ARI within the different sources of data (primary health care, district and University Hospitals )
□	Absence of a systematic microbiological surveillance for the etiology of ARI in the hospital setting
□	Generalized absence of data for the etiology of non-severe ARI within the community
□	Poor assessment of viral etiology of ARI
□	Lack of evidence on the role of air pollution as a contributor to the incidence and the severity of ARI
□	Lack of investigation on the social determinants of ARI and the access to care
□	Evidence-based data is produced depending on the focus of interest of the clinical wards and the research laboratories.
□	There is no evaluation or control of the effectiveness and adequacy of the antibiotics prescribed for ARI
□	The local risk factors for ARI have been poorly explored.
□	The epidemiological indicators which could permit the study of the impact of the introduction of conjugate pneumococcal vaccines are not collected
□	Lack of a strategic long term ARI plan at the research and policy level.

Additionally, authors of the different papers, have used different case definition, or involved different study populations. Data produced by hospitals, and in particular that of the University Hospital of Casablanca, arise from local laboratories offering microbiological point monitoring data of patients admitted to the clinical services of their structure. While these local data are not representative of the rest of the Moroccan population under 5 years of age, the evident scarcity of available data is the reason behind its usefulness to guide the countries’ first line treatment against ARI, or similarly, the desired serotype composition of the newly introduced pneumococcal conjugate vaccine. Better, more geographically representative, detailed etiological and epidemiological data of the principal pathogens responsible for paediatric ARI are urgently needed, so as to base in evidence current recommendations for its prevention and management.

The mortality rate of ARI reported by the national health statistics in 2010, among children less than one year old (2,5%) is similar to the mortality rate reported globally (2,3%) and in developing countries (2,4%) [6]. The same report estimates that 4% of death occurs within a hospital setting. However, these statistics, like in many other countries, should be interpreted with caution, as the sources from which they are gathered may vary, and because death certificates are not filled uniformly, leading to potential over or underestimating the IRA as causes of death.

In Morocco, ARI-associated mortality statistics do not seem to show significant differences between boys and girls, such as those observed in many Asian countries [6].

The description of the clinical patterns of ARI throughout the last of 17 years shows a changing profile of the reported types of cases. More than representing a real epidemiological transition, sporadic reports seem to translate the particular focus or scientific interests of the team of the ward where the data were extracted. Needless to say, they do represent an important reporting bias.

The laboratory studies are few in numbers, are recent, and are unrelated, using different methods, different populations of study and different specimens’ types. As a result, they are difficult to compare, also, and trends in antimicrobial resistance are challenging to infer. The laboratory of Casablanca seems to be the single reference laboratory where long-term surveillance of ARI has been implemented, and has been providing aggregate data since the year 1999. This essentially microbiological surveillance, relatively basic at the moment, should be optimized by including data on patient’s characteristics, their clinical manifestations, the prescribed treatments and outcomes, so as to better describe the burden of microorganisms isolated and the population at risk. Furthermore, a careful monitoring should also be conducted in the pilot settings where IMCI is implemented to follow trends of the antimicrobial sensitivity patterns, particularly in the light of the suspected overuse of antibiotics, as antibiotic prescription seems to be also very common practice. Such data could provide evidence-based orientation among the identity of antibiotics recommended through IMCI, their dose adjustments, or a possible need for replacement.

Only one recently published study documents the complete picture of clinical severe pneumonia among admitted children in a university setting, by collecting individual data, related to the demographic status, clinical and biochemical characteristics, microbiological patterns and prognosis factors and the evolution of the disease [15]. The limitation of this study is that its results cannot be extrapolated to the entire country, because this area is better than average covered in terms of health infrastructures, and is a predominantly urban.

Regarding the pneumococcal conjugate vaccine, the lack of consistent and geographically representative data on the circulating serotypes known to cause paediatric invasive bacterial disease in Morocco hampers our understanding of the potential effectiveness of the introduction of such vaccine. It would appear essential to monitor prospectively what pneumococcal serotypes are still causing disease among Moroccan children, not only to understand whether this pathogen remains the major public health concern, but also demonstrate whether serotype replacement is really occurring [34].

The risk factors identified among admitted children with ARI are common to those documented in other settings [35]. Risk factors identified among children in a hospital setting were related to the management of the disease, and also relate to the need of the improvement of the quality of care of patients admitted for ARI. Among these risk factors an easily preventable one relates to control tobacco exposure. One study reported that passive smoking is a real concern as a risk factor for ARIs in Morocco, having this country still not ratified the convention on the tobacco control [36]. Therefore, such data can be used as a strong argument to help the scientific community lobby in the activation of this process for the protection of the health of children and citizens in general.

The few available data sources that we have been able to identify, may adequately describe local patterns of ARI, but clearly have important limitations as to their use to describe the burden of ARI at national level or their extrapolation to the general population to estimate the burden of disease or epidemiological trends. Moreover, knowledge gaps found of ARI in Morocco identified by this review seem to be common to other developing countries [4,7], where the real burden and impact of childhood ARI and/or pneumonia is difficult to assess and to document.

**Figure 5:**
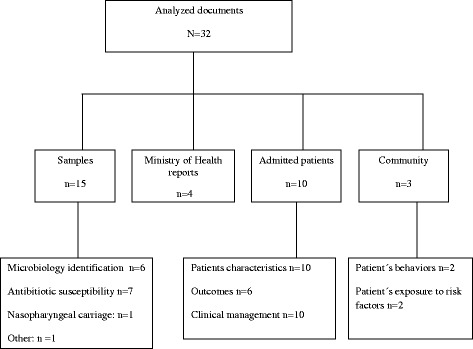
Main information identified in the documents included in the systematic review analysis of ARI among children in Morocco. From 1997 to 2014.

To our knowledge, this is the first review that has collected evidence from a variety of sources of data for ARI in Morocco. This is a critical step which has allowed identifying and summarizing already existing relevant data, (Figure 5), but also shows the weaknesses of the current national surveys designed to estimate the real burden of ARI caused by *Streptococcus pneumoniae* and to monitor the antibiotic resistance among the prescribed antibiotics for ARI.

Morocco should promptly develop a set of common indicators for tracking progress in its fight against pneumonia and use these data to identify groups at greater risk or missed by Health care Services and develop integrative approaches to reach them.

## Conclusions

This review confirms the scarcity and limitations of the available epidemiological, clinical, and microbiological data regarding ARIs in Morocco, without which evidence based-management and preventive strategies are difficult to implement. Efforts should be made towards the development of research strategies on all the fields of social health determinants of ARIs, to permit a holistic approach for understanding the determinants of ARI in Morocco and establishing adequate control measures. Additionally, it would be desirable that Moroccan researchers attempt to disseminate more widely the data they generate so that it reaches the wider scientific community and can be used to provide reliable indicators for national and international policy makers to guide future preventives and therapeutic strategies based on solid evidence.
